# Genome-Wide Identification of *APX* Gene Family in *Citrus maxima* and Expression Analysis at Different Postharvest Preservation Times

**DOI:** 10.3390/genes15070911

**Published:** 2024-07-12

**Authors:** Yu Zhang, Yujiao Peng, Huixin Zhang, Qiuyu Gao, Fangfei Song, Xueyu Cui, Fulei Mo

**Affiliations:** 1 Key Laboratory of Beibu Gulf Environment Change and Resources Utilization of Ministry of Education, Nanning Normal University, Nanning 530001, China; 2 College of Life Sciences, Northeast Agricultural University, Harbin 150030, China; 3 Guangxi Key Laboratory of Earth Surface Processes and Intelligent Simulation, Nanning Normal University, Nanning 530001, China

**Keywords:** *Citrus maxima*, *APX* gene family, reactive oxygen species, postharvest preservation, ascorbic acid

## Abstract

Ascorbate peroxidase (APX) is a crucial enzyme involved in cellular antioxidant defense and plays a pivotal role in modulating reactive oxygen species (ROS) levels under various environmental stresses in plants. This study utilized bioinformatics methods to identify and analyze the *APX* gene family of pomelo, while quantitative real-time PCR (qRT-PCR) was employed to validate and analyze the expression of *CmAPXs* at different stages of fruit postharvest. This study identified 96 members of the *CmAPX* family in the entire pomelo genome, with uneven distribution across nine chromosomes and occurrences of gene fragment replication. The subcellular localization includes peroxisome, cytoplasm, chloroplasts, and mitochondria. The *CmAPX* family exhibits a similar gene structure, predominantly consisting of two exons. An analysis of the upstream promoter regions revealed a significant presence of cis-acting elements associated with light (Box 4, G-Box), hormones (ABRE, TCA-element), and stress-related (MBS, LTR, ARE) responses. Phylogenetic and collinearity analyses revealed that the *CmAPX* gene family can be classified into three subclasses, with seven collinear gene pairs. Furthermore, *CmAPXs* are closely related to citrus, pomelo, and lemon, followed by Arabidopsis, and exhibit low homology with rice. Additionally, the transcriptomic heat map and qPCR results revealed that the expression levels of *CmAPX57*, *CmAPX34*, *CmAPX50*, *CmAPX4*, *CmAPX5*, and *CmAPX81* were positively correlated with granulation degree, indicating the activation of the endogenous stress resistance system in pomelo cells by these genes, thereby conferring resistance to ROS. This finding is consistent with the results of GO enrichment analysis. Furthermore, 38 miRNAs were identified as potential regulators targeting the *CmAPX* family for post-transcriptional regulation. Thus, this study has preliminarily characterized members of the *APX* gene family in pomelo and provided valuable insights for further research on their antioxidant function and molecular mechanism.

## 1. Introduction

Reactive oxygen species (ROS), as by-products of cell respiration, protein folding, and many metabolic reactions, are produced by plant metabolism from chloroplasts, microbodies, and other organelles or plasma membrane electron transport pathways under normal environmental conditions. Low concentrations of ROS play an important role in regulating the cell cycle, signal transduction, stomatal conductance, and maintenance of the photosynthetic system [1,2,3]. After plants are subjected to stress, the intracellular ROS balance is destroyed, and a large amount of ROS accumulation leads to the damage of proteins, DNA, and other components and the destruction of the photosynthetic system, which affects the normal growth and development and the metabolic process of plants [4,5]. In order to resist the damage of adverse environmental conditions, plants have gradually evolved two kinds of enzyme-promoted and non-enzymatic defense mechanisms for self-protection, among which enzymatic reactions mainly include ascorbate peroxidase (APX), catalase (CAT), glutathione peroxidase (GPX), and other antioxidant enzymes [6].

APX is one of the most important antioxidant enzymes in higher plants and has a high specificity and affinity for H_2_O_2_. Using ascorbic acid as an electron donor, *APX* catalyzes the conversion of H_2_O_2_ into H_2_O and O_2_ through the ascorbate–glutathione cycle (ASA-GSH) to generate monodehydroascorbate so as to reduce or even avoid the damage caused by the excessive accumulation of reactive oxygen species under stress and maintain the intracellular ROS balance [7]. *APX* belongs to a multigene family, which encodes a series of isoenzymes of different organelles and is divided into four types according to their subcellular localization, cytoplasmic type (*cAPX*), chloroplast type (*chlAPX*), mitochondrial type (*mitAPX*), and microtype (*mAPX*), and the number varies among different species [8]. Eight kinds of *APX* were found in *Arabidopsis thaliana*, comprising three cytoplasmic *APX*, two chloroplast *APX*, and three *microAPX* [9]. The number of *APX* genes in model crops such as rice, tomato, pepper, and watermelon fluctuated between 5 and 8 genes, while 10 *APX* genes were found in maize and 166 *APX* genes were found in peanuts, which appeared to be the result of gene replication events [10,11]. Notably, *AtAPX4* does not contain catalytic residues of the ascorbic acid and heme-binding site, so it has been renamed *APX-L* [12].

The accumulation of excess ROS affects the growth and development of plants and even causes plant death. The removal of excess ROS in plants is related to the strength of plant stress resistance. *APX* plays a central role in the plant reactive oxygen scavenging network and is a key factor regulating the intracellular redox state. The relationship between *APX* and plant resilience has been studied in many plants [13]. In rice, the overexpression of *OsAPX2* can enhance plant tolerance to drought, salt, and low-temperature stress, while the knockout of the *APX2* gene can damage plant development and growth [14]. Compared with wild-type sweet potato plants, transgenic sweet potato plants with the *APX* gene in pea showed significantly better salt tolerance and faster root growth and development [15]. The overexpression of the *PutAPX* gene enhanced ROS clearance, thereby enhancing salt tolerance in *Puccinellia tenuiflora* [16]. Nine *APXs* and seven *GPXs* were identified in sorghum, and RNA-seq and qRT-PCR analysis showed that *APX*/*GPX* genes were significantly regulated under drought stress [17]. Under low-temperature stress, the overexpression of the *AnAPX* gene reduced the electrolyte leakage rate (EL) and malondialdehyde (MDA) content of tobacco, thereby increasing the plant cold resistance [18]. However, the effect of *APX* on the resistance of pomelo to oxidative stress has not been reported.

*Citrus maxima* is a citrus plant in the rutaceae family. It has a large fruit type, a unique flavor, a sweet fragrance, and excellent storage and transportation capacity, and vitamin C content is the major component of citrus, which is favored by the majority of consumers [19]. L-ascorbic acid (AsA), also known as vitamin C, is the main antioxidant and an important secondary metabolite in pomelo and plays an important role in its cell growth, metabolism, and aging process. AsA can use the ASA-GSH to indirectly remove hydrogen peroxide, thus protecting the pomelo organism and its normal metabolism from the damage caused by oxidative stress [20]. Therefore, pomelo is a valuable material for studying the cloning and regulation of key genes in ascorbic acid biosynthesis and metabolism pathways [21]. In actual production, fruit granulation is a physiological disease during maturity and the postharvest storage period. During postharvest storage, the juice sacs become dry and shriveled, often coexisting with preharvest granulation in the same fruit. In severe cases, the color of the juice sacs becomes earthy, and the spongy layer of the peel becomes loose. This directly results in a significant decline in fruit quality and storage performance [22,23]. In this study, the *CmAPX* gene family of pomelo was identified at the whole gene level, and its evolutionary relationship, gene structure, conserved domain, promoter cis-acting elements, tissue expression, and the correlation between APX, active oxygen free radical scavenging, and juice granulation were analyzed, which provides a theoretical foundation for the further analysis of the role of *APX* gene family members in the storage process of pomelo to enhance the quality and yield of pomelo fruit.

## 2. Materials and Methods

### 2.1. Plant Materials and Treatment

Pomelo (*C. maxima* (Burm. f.) Merr) was selected from an orchard in Xingping Town in Guilin Country (24°46′50.8″ N, 110°29′32.1″ E), Guangxi Province. The test trees were old pomelo trees with an age of 30 years in the orchard. When the fruits were ripe, pomelo pulp stored for 0 d, 20 d, 40 d, and 60 d was selected for total RNA extraction; total RNA was extracted with a plant RNA mini kit (Watson, Shanghai, China).

### 2.2. Identification and Characterization of the APX Gene Family Members in Pomelo

The TAIR from the *A. thaliana* genome database (https://www.arabidopsis.org (accessed on 1 April 2024) was utilized to access the sequences of 8 *AtAPX* genes, which were then subjected to BLASTP analysis using the pomelo genome sequence (*Citrus grandis* (L.) Osbeck. cv. ‘Wanbaiyou’ v1.0, http://citrus.hzau.edu.cn/index.php (accessed on 1 April 2024). Additionally, the protein family database Pfam (http://pfam.xfam.org/ (accessed on 1 April 2024) was employed to search for the hidden Markov model HMM of the *APX* gene family. Furthermore, NCBI databases CDD (https://www.ncbi.nlm.nih.gov/Structure/cdd/cdd.shtml (accessed on 3 April 2024), SMART (http://smart.embl-heidelberg.de (accessed on 3 April 2024), and other online software were used for selection and verification to ensure that the *CmAPX* protein sequence contains the ascorbate peroxidase domain (PF00141). The final results revealed a total of 96 identified *CmAPX* genes in the pomelo genome through BLASTP and HMM analyses [24,25]. Moreover, Expasy (https://web.expasy.org/protparam/ (accessed on 5 April 2024) was utilized to predict physical and chemical properties such as isoelectric point, molecular weight, number of amino acids, and other related information for *CmAPX* amino acid sequences [26]. Subcellular localization predictions for *CmAPX* proteins were conducted using the CELLO v.2.5 website [27].

### 2.3. Chromosomal Localization Analysis of the APX Gene Family in Pomelo

The *CmAPX* chromosome location data were retrieved from the PGR database, and TBtools was utilized for gene mapping on chromosomes. Phylogenetic trees were constructed to determine the evolutionary relationships of *APX* proteins among pomelo, Arabidopsis, rice, and citrus. Multiple sequence alignment of *APX* proteins was conducted using the MEGA 7.0 software. Additionally, a phylogenetic tree with 1000 bootstrap repeats was generated using the neighbor-joining (NJ) method. iTOL was employed for enhancing the visual presentation of the phylogenetic trees [28].

### 2.4. Phylogenetic Relationships and Collinearity Analysis of the APX Gene Family in Pomelo

The homology analysis of *APX* genes among pomelo, citrus, Arabidopsis, and rice was conducted using the MCScanX tool and visualized with the Advance Circos package in TBtools software (v2.096) [29]. Additionally, multicollinearity analysis of the *APX* gene was performed using the multiple syntenyPlotteR package in TBtools software (v2.096). Furthermore, the Ka/Ks ratio of all *CmAPX* was predicted using the simple Ka/Ks calculator in TBtools software (v2.096) [30].

### 2.5. Analysis of Conserved Motif and Gene Structure of APX Gene Family in Pomelo

The TBtools software was utilized for the identification of the protein-coding region (CDS, coding sequence) and non-translation region (UTR, untranslated region) of *CmAPXs* [31]. The protein sequence of pomelo with *APX* gene family members was uploaded to the MEME online website for analysis. The parameters were set as follows: the number of conserved identification motifs is 10, the amino acid length of the smallest motif is 6, the amino acid length of the largest motif is 100, and the number of searches is 10,000. Subsequently, TBtools software was employed to visualize the conserved motifs and gene structures of these genes [32].

### 2.6. Analysis of Cis-Acting Elements of APX Gene Family in Pomelo

To predict the cis element of the *CmAPX* promoter, the upstream sequence of 2 kb from the initiation codon of all *CmAPX* genes isolated from the pomelo genome was analyzed using PlantCARE (http://bioinformatics.psb.ugent.be/webtools/plantcare/html (accessed on 9 April 2024), and visual analysis was performed using TBtools software [33].

### 2.7. Prediction of miRNA-Targeting CmAPX Gene and Analysis of GO Function Annotation

The psRNATarget website was utilized to predict the target sites of miRNA for *CmAPX* CDS targeting, and the interaction network diagram between miRNA and *CmAPX* was generated using Cytoscape software (v3.10.2) [34]. Gene ontology (GO) was employed to annotate all *CmAPX* protein sequences, and the enrichment results were visually analyzed with TBtools software [35].

### 2.8. Analysis of APX Gene Expression in Pomelo Based on Transcriptome Data

To analyze the expression pattern of *APX* during pomelo juice sac development, the transcriptome data (accession number PRJNA817805) were used to obtain the expression levels of all *APX* genes in pomelo fruit near the core (NC) and far away from the core (FC) at 157 days (stage 1, S1), 180 days (stage 4, S4), and 212 days (stage 8, S8) after flowering by using TBtools software. Subsequently, TBtools software was utilized to generate a circular heat map [36].

### 2.9. The Expression Patterns of the APX Gene at Different Storage Times Were Analyzed Using qRT-PCR

The first strand of cDNA was synthesized using an M5 Super plus qPCR RT kit with gDNA remover from Keyi Biotechnology Co., Ltd. (Shanghai, China). The primers were designed using the Primer Premier 5 software and synthesized by Beijing Tsingke Biotechnology Co., Ltd. (Supplementary Table S1). *Actin* was selected as the reference gene. The reaction system was 2× Realtime Super mix at 10 μL, forward and reverse primer concentration at 10 nmol·L^−1^, cDNA at 1.0 μL, and ddH_2_O at 8 μL. We set up 3 biological replicates. The PCR instrument model was the CFX96TM Real-Time System. The reaction procedure of qRT-PCR was as follows: 95 °C for 1 min, 95 °C for 10 s, 60 °C for 5 s, and 72 °C for 12 s, for a total of 40 cycles. The relative gene expression was calculated with 2^−∆∆Ct^ [37]. Excel 2019 was used to sort out the experimental data, and GraphPad Prism 9 was used to analyze the difference significance. A heat map of gene expression was constructed using TBtools.

## 3. Result

### 3.1. Identification of APX Gene Family in Pomelo

**Figure 1:**
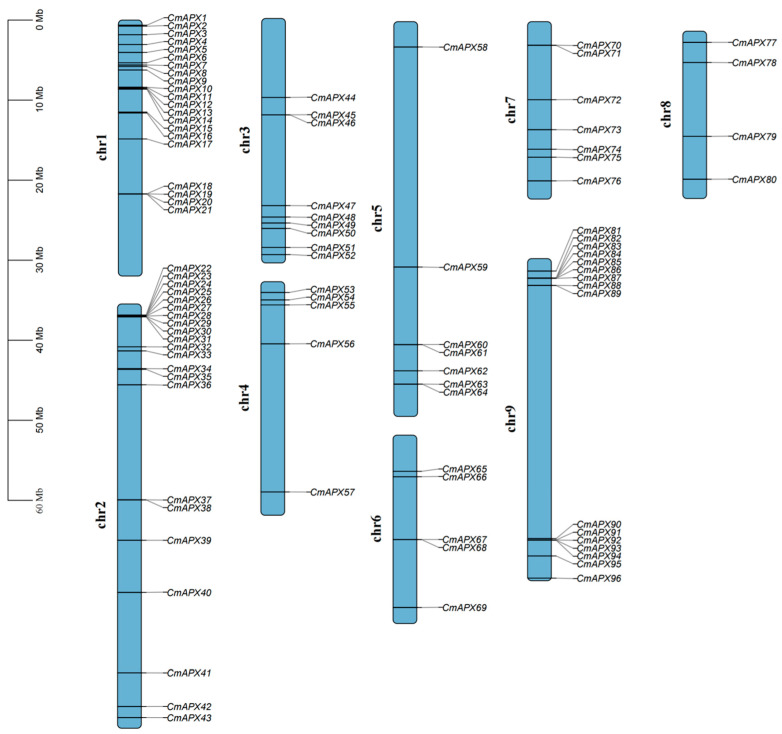
Chromosome location of the *APX* gene in pomelo; the bar scale on the left indicates chromosome length.

According to the whole-genome data of pomelo, members of the *APX* gene family were identified using HMMER 3.0 (PF00141) and further confirmed using the CDD, Pfam, and SMART websites (accessed on 3 April 2024). A total of 96 *CmAPXs* were identified and unevenly distributed on nine chromosomes, named *CmAPX1* to *CmAPX96* in turn. Chromosomes 1, 2, and 9 harbored a higher number of genes, with 21, 22, and 16, respectively, while only 4 genes were found on chromosome 8. Additionally, gene aggregation on chromosome 1 is located near the centromere in the middle of the chromosome, whereas gene distribution on chromosomes 2, 4, and 9 is situated at both ends away from the centromere region (Figure 1). The physical and chemical properties of 96 *CmAPX* genes were further analyzed. The results revealed significant variation in the gene length of *APX* family members, with coding region lengths ranging from 318 to 3219 bp, protein lengths ranging from 106 to 1073 amino acids, and molecular weights of the encoded proteins ranging from 11,484.94 to 116,743.82 Da. Additionally, the isoelectric points ranged between 4.43 and 10. Subcellular localization prediction indicated that 13 *CmAPX* genes were located in the cytoplasm, 4 in lysosomes, 1 in mitochondria, 2 in the nucleus, and 5 in chloroplasts. The diverse subcellular localization of *APX* isoenzymes enables them to regulate reactive oxygen species levels within different cellular compartments, thereby protecting plants from oxidative damage (as shown in Supplementary Table S2).

### 3.2. Phylogenetic Analysis of APX Proteins

**Figure 2:**
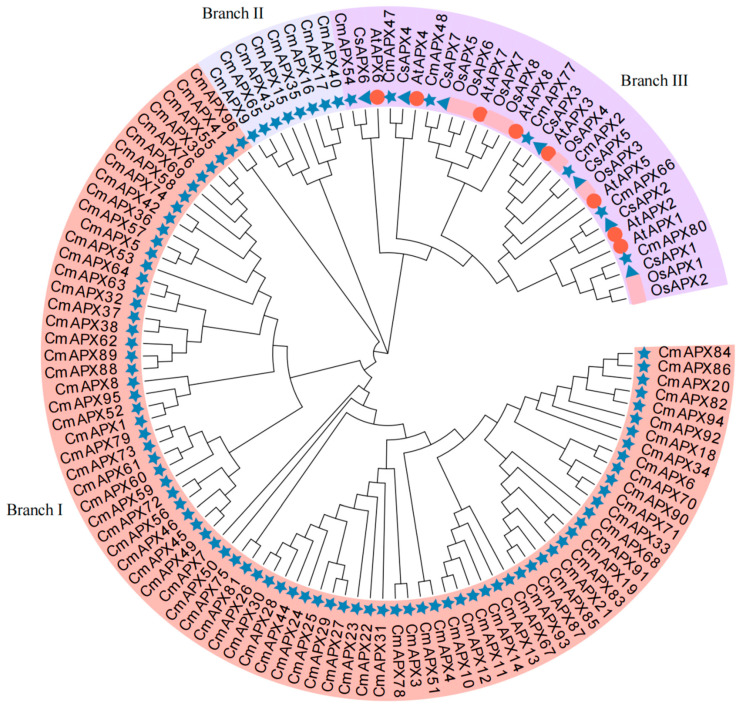
Phylogenetic analysis of the amino acid sequence of APX from *C. maxima* (five-pointed star), *C. sinensis* (triangle), *A. thaliana* (circle), and *O. sativa* (square).

The evolutionary relationship of APX family proteins in pomelo was studied by constructing a phylogenetic tree based on a multiple sequence comparison between APX members of pomelo and homologous proteins of Arabidopsis, rice, and citrus (Figure 2). The three APX proteins can be divided into three branches, in which Branch I and Branch II contain 81 CmAPXs and 8 CmAPXs, respectively, and Branch III contains 7 CmAPXs, 8 AtAPXs, 8 OsAPXs, and 7 CsAPXs. In addition, 7 CmAPXs and 7 CsAPXs in Branch III can all form parallel homologous pairs, such as CmAPX54 and CsAPX6, CmAPX47 and CsAPX4, CmAPX48 and CsAPX7, CmAPX77 and CsAPX3, CmAPX2 and CsAPX5, CmAPX66 and CsAPX2, and CmAPX80 and CsAPX1, suggesting that CmAPXs and CsAPXs in this clade are highly conserved in evolution and may have similar functions in pomelo and citrus.

### 3.3. Analysis of Conserved Motif and Gene Structure of CmAPXs

**Figure 3:**
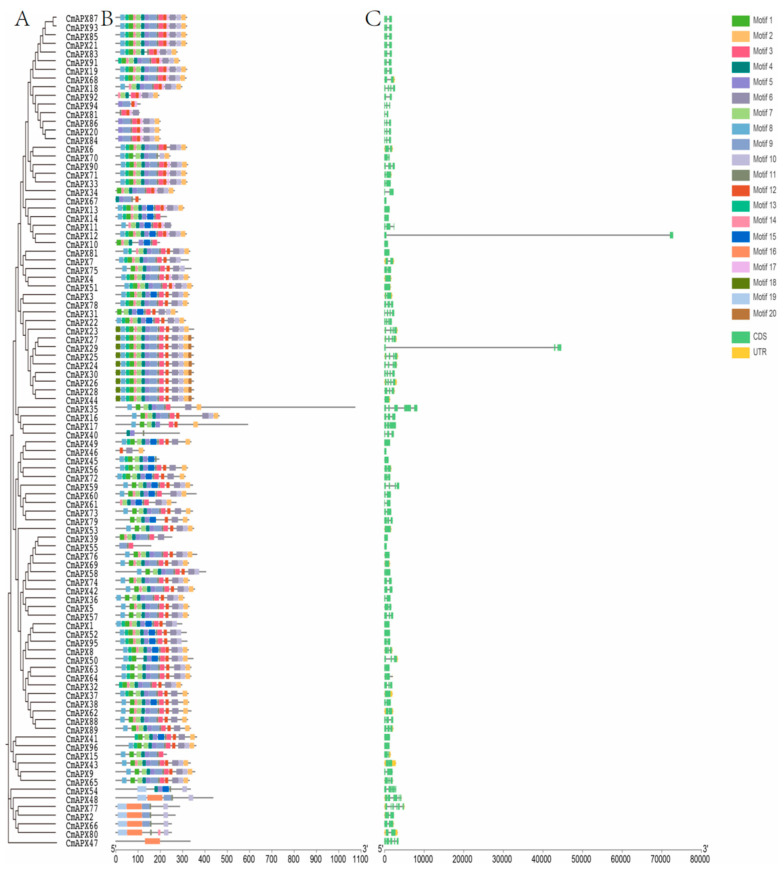
Gene structures and conservative motif analysis of the *APX* gene family in pomelo. (**A**) phylogenetic tree of CmAPX proteins; (**B**) conserved motifs of CmAPX proteins; (**C**) gene structure of *CmAPX* genes.

The MEME (v5.5.4) and TBtools software were utilized for the prediction of conserved motifs within the *CmAPX* gene family (Figure 3B). The findings revealed that Motif 6, Motif 10, and Motif 12 were present in the majority of CmAPX protein structures, indicating their status as the most highly conserved domains within the pomelo *APX* gene family. Furthermore, paralogous gene pairs exhibited identical numbers and sequences of conserved protein motifs; notably, CmAPX87, CmAPX93, CmAPX85, CmAPX21, CmAPX68, and CmAPX19 shared 15 identical conserved motifs. Similarly, CmAPX27, CmAPX29, CmAPX25, CmAPX24, CmAPX30, CmAPX26, CmAPX28, and CmAPX44 contained sixteen identical conserved motifs, while CmAPX77, CmAPX2, and CmAPX66 had three identical conserved motifs with consistent ordering. Conversely, CmAPX47, which was grouped separately, contained the lowest number of conserved motifs, with only one conserved motif. Consequently, Motif 6, Motif 10, and Motif 12 are postulated to represent crucial domains within the *APX* gene family; moreover, the proteins encoded by genes from the same subfamily exhibit high similarity in terms of type, number, and arrangement of their respective conserved motifs (Figure 3A).

The analysis of the exon and intron structure (Figure 3C) revealed that the majority of genes contained between one and six exons, with *CmAPX48* and *CmAPX77* having the highest number (six). Conversely, 23 genes, including *CmAPX67*, *CmAPX13*, and *CmAPX14*, had the lowest number of exons at only one. Consequently, most members of the *CmAPX* gene family contained between one and two introns, with *CmAPX48* and *CmAPX77* having the highest number of introns at five. Despite similar coding sequence lengths, there is significant variation in gene full length due to differences in intron length within this gene family during evolution. This may contribute to functional diversity and serves as a valuable reference for further investigation into evolutionary relationships among different subfamilies within the pomelo *APX* gene family.

### 3.4. Analysis of Promoter Cis-Acting Elements of CmAPX Gene Family Members

**Figure 4:**
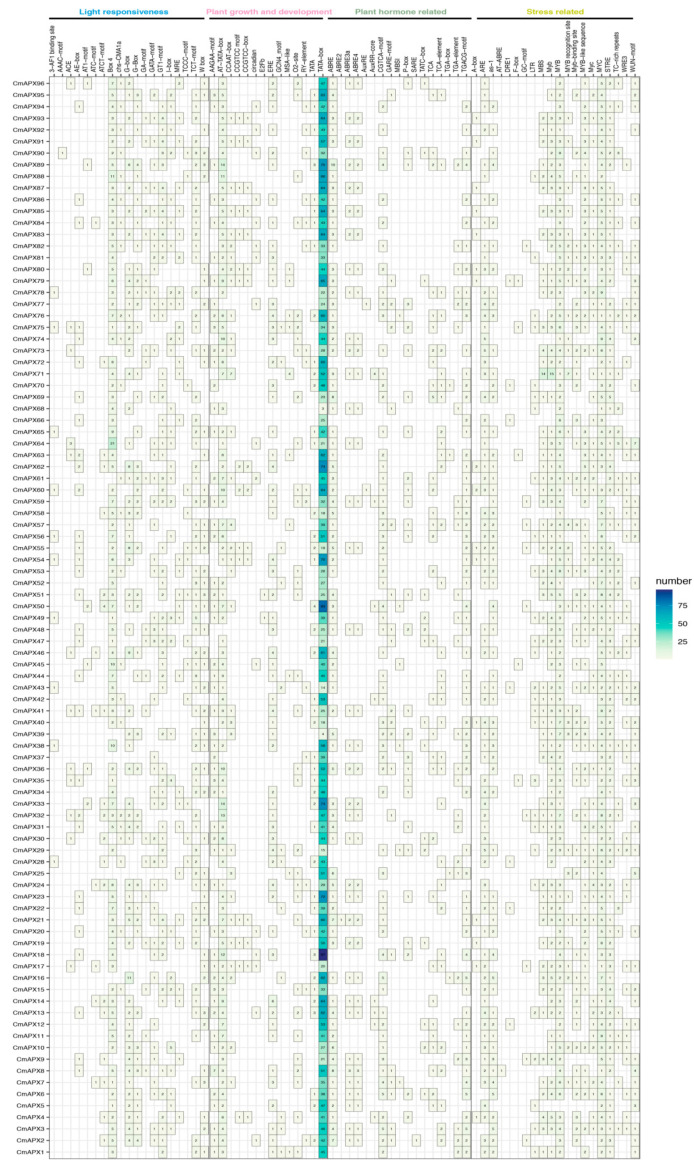
Analysis of the cis-acting elements of the *APX* gene promoter in pomelo. The depth of color in the figure represents the amount, with light green colors indicating a smaller or zero quantity, and dark green colors indicating a larger quantity.

The promoter is a cis-acting element associated with the initiation of gene expression and is an important component of structural genes (Figure 4). A total of 70 cis-acting elements were identified in the initiation regions of *CmAPX* gene family members, which could be classified into four categories: light responsiveness, plant growth and development, plant hormone-related elements, and stress-related elements. Among them, a total of 19 cis-acting elements were identified as light-responsive, namely, 3-AF1 binding site, AAAC-motif, ACE, AE-box, AT1-motif, ATC-motif, ATCT-motif, Box 4, chs-CMA1a, G-box, G-Box, GA-motif, GATA-motif, GT1-motif, I-box, MRE, CTC-Motif, CTT-Motif, and W-box. We found that, except for 8 *CmAPXs*, all other genes contained Box 4, and even CmAPX64 contained 21. There are also 14 cis-acting elements associated with plant growth and development: AAGAA motif, AT-TATA-box, CCAAT-box, CCGTCC motif, CCGTCC box, circadian, E2Fb, ERE, GCN4_motif, MSA-like, O2-site, RY-element, TATA, and TATA-box. The promoters of the 96 *CmAPXs* all contain typical TATA-box core cis-acting elements, while AAGAA motif, AT-TATA-box, and ERE are present in most *APX* gene members.

The promoter structure of *CmAPX* generally consists of five types of hormone response elements, namely, the MeJA response element (CGTCA/TGACG-motif), gibberellin response element (TATC/P/ARE-motif), ABRE response element (ABRE), salicylic acid response element (TCA-element), and auxin response element (TGA-element/AuxRR-core). Specifically, the CGTCA/TGACG-motif is present in the promoter regions of 62 *CmAPX* members, with *CmAPX3*, *CmAPX6*, and *CmAPX16* having the highest number of promoter regions at 5. The TATC/P/ARE-motif is found in the promoter regions of 28 *CmAPX* members. ABRE is the most common among the promoters of *CmAPX* gene family members, with *CmAPX89* containing the most promoter regions at 10. There are between one and two TCA-elements in 32 members’ promoter regions. The TGA-element/AuxRR-core exists in the promoter regions of 26 *CmAPX* members, with *CmAPX71* having the most at 4.

Almost all *CmAPX* genes contain at least one element associated with stress response in their promoter region. These elements include low-temperature response elements (LTR), anaerobic inducible elements (ARE, GC-motif), drought inducible elements (MBS), defense and stress response elements (TC-rich repeats), and wound response elements (WUN-motif). These findings suggest a close association between the *CmAPX* gene family members and various stress responses involved in pomelo stress resistance. Specifically, MBS is present in 56 promoter regions of *CmAPX* members, with 14 instances found in *CmAPX71*. LTR has been identified in the promoter region of 27 *CmAPX* members. With the exception of *CmAPX35*, which has three promoter regions, the remaining 26 genes each contain only one to two such elements. TC-rich repeats are present in 33 promoter regions of *CmAPX* members, with the highest number observed in *CmAPX64* and *CmAPX22*, containing 5 and 3, respectively. Other cis-elements such as WUN-motifs are found in the promoter region of *CmAPX64* for a total of seven occurrences; MYB binding sites are absent only from the promoter regions of *CmAPX28*, *CmAPX25*, and *CmAPX5*; AT-ABRE is exclusively present solely within the promotor region of *CmAPX8*.

### 3.5. Tandem Gene Duplication and Segmental Gene Duplication of CmAPXs

The Ka/Ks ratio, which compares non-synonymous substitutions (Ka) to synonymous substitutions (Ks), is a crucial determinant of selection pressure on protein-coding genes and holds significant importance in evolutionary analysis. In this study, the Ka/Ks values were analyzed for 96 *CmAPX* gene-coding regions in pomelo (Supplementary Table S3). It was found that the Ka/Ks ratios between *CmAPX20* and *CmAPX92*, *CmAPX84* and *CmAPX92*, as well as *CmAPX86* and *CmAPX92* exceeded 1, indicating positive selection effects on these gene members. Conversely, the Ka/Ks values for other *CmAPX* gene members were less than 1, suggesting strong purifying selection during their evolutionary history and potential functional similarity among these gene pairs. Additionally, in the analysis of *CmAPX* gene tandem replication events, we observed a linear relationship between six tandem replicators (*CmAPX77* and *CmAPX2*, *CmAPX78* and *CmAPX3*, *CmAPX52* and *CmAPX1*, *CmAPX51* and *CmAPX4*, *CmAPX68* and *CmAPX18*, *CmAPX66* and *CmAPX88*), with high sequence similarity. These replicators can contribute to genetic diversity in the genome through changes in gene copy number and structural differences, thereby influencing organism growth and development, trait expression, genetic variation, and other life activities.

**Figure 5:**
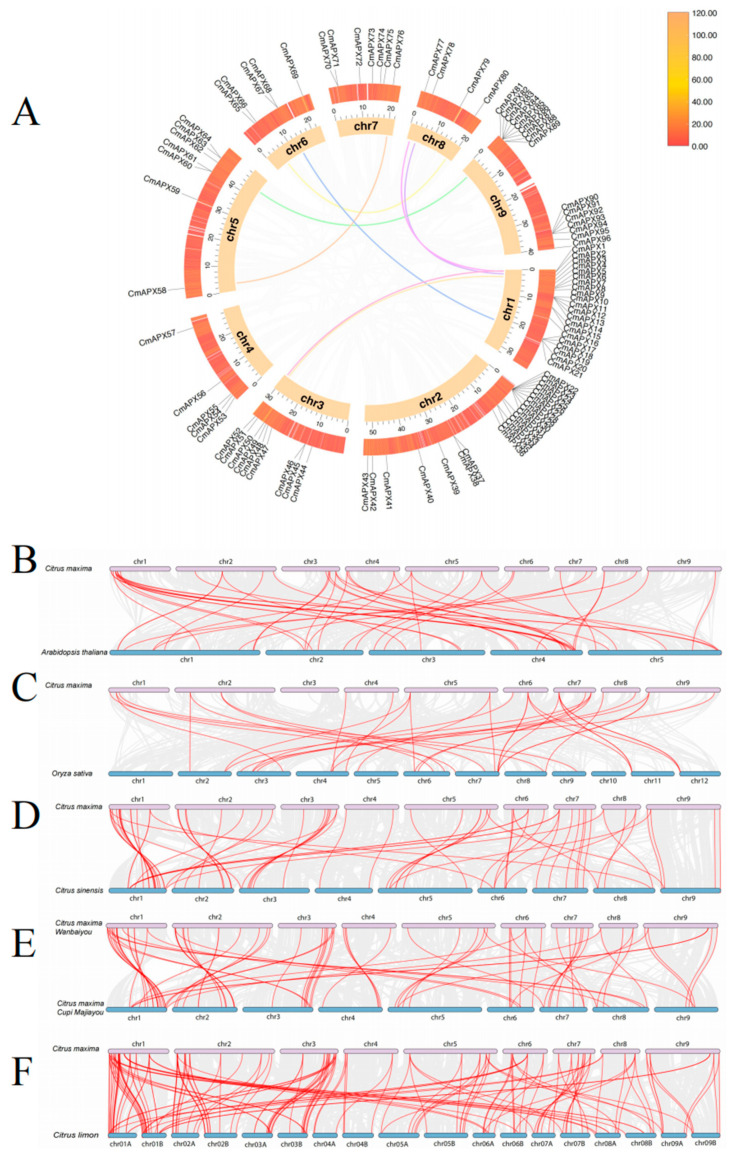
Collinearity analysis of *APX* genes in *C. maxima* (**A**) and between *C. maxima and A. thaliana* (**B**), *O. sativa* (**C**), *C. sinensis* (**D**), *C. maxima* cv Cuipi Majiayou (**E**), and *C. limon* (**F**). Genes of *APX* subfamilies with gene duplication relationships are connected by lines in red.

To further investigate the genetic correlation among *APX* gene family members, we conducted an analysis of collinearity involving pomelo, for both the reference genome cv. Wanbaiyou (Figure 5A) and another cv. Cuipi Majiayou (Figure 5E), *A. thaliana* (Figure 5B), rice (Figure 5C), citrus (Figure 5D), and lemon (Figure 5F). The results revealed that 46 *CmAPX* genes exhibited collinearity with Arabidopsis genes, 36 *CmAPX* genes showed collinearity with rice genes, 68 *CmAPX* genes displayed collinearity with citrus genes, 79 *CmAPX* genes demonstrated collinearity with pomelo genes, and 156 *CmAPX* genes were found to be collinear with lemon genes. These findings indicate a large number of conserved regions and homologous gene pairs within the genomes of citrus, pomelo, and lemon in the same genus. Furthermore, there is evidence of gene replication and conservation during the evolutionary process of this gene. Therefore, it can be concluded that pomelo *CmAPX* is most closely related to citrus, pomelo, and lemon, followed by Arabidopsis; however, it exhibits low homology with *OsAPXs* members. Additionally, some of the genes were found to be collinear with at least two pairs of homologous genes; in particular, *Cg1g008440.1*, *Cg1g006370.1*, *Cg1g001450.1,* and *Cg2g018020.1* in pomelo had multiple homologous copies in Arabidopsis, rice, citrus, and lemon. Therefore, it can be inferred that whole duplication events may have occurred during the evolution of the *APX* gene family.

### 3.6. Functional Characterization of the APX Gene Family Members in Pomelo

**Figure 6:**
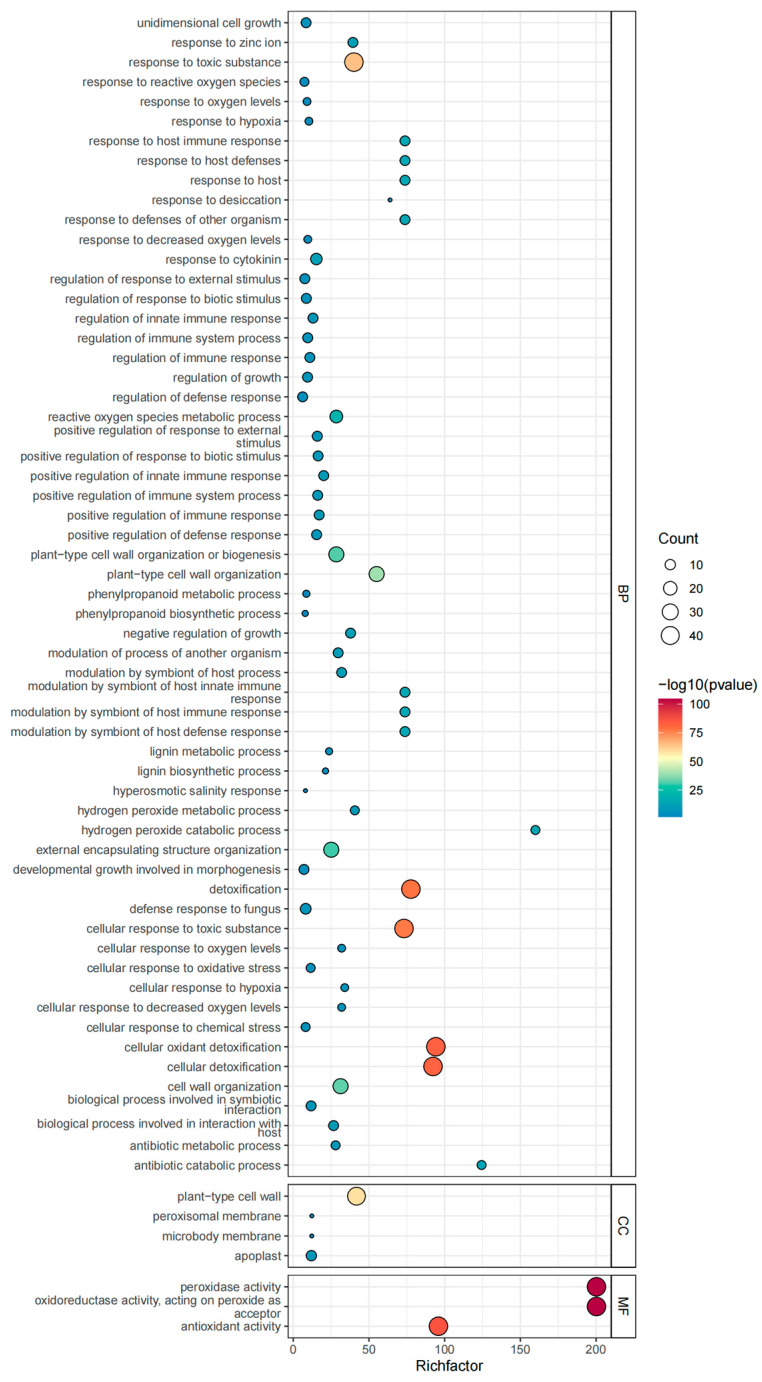
CmAPX GO function classification diagram.

The pomelo APX proteins were classified with GO functional annotation (Figure 6), resulting in their categorization into three groups: biological process, cellular component, and molecular function. Molecular function accounted for the least, and the annotation results of biological processes and cell components accounted for the most. Specifically, the biological processes mainly focused on cellular oxidant detoxification, cellular detoxification, and cellular response to toxic substance articles. Cell components were primarily concentrated in peroxidase activity, oxidoreductase activity acting on peroxide as acceptor, and antioxidant activity. Furthermore, a higher number of genes with molecular functions was associated with plant-type cell wall entries. This suggests that APX plays a crucial role in H_2_O_2_ metabolism within pomelo cells as a key component of the hydrogen peroxide detoxification system.

### 3.7. Analysis of Gene Regulatory Network of APX and miRNA

**Figure 7:**
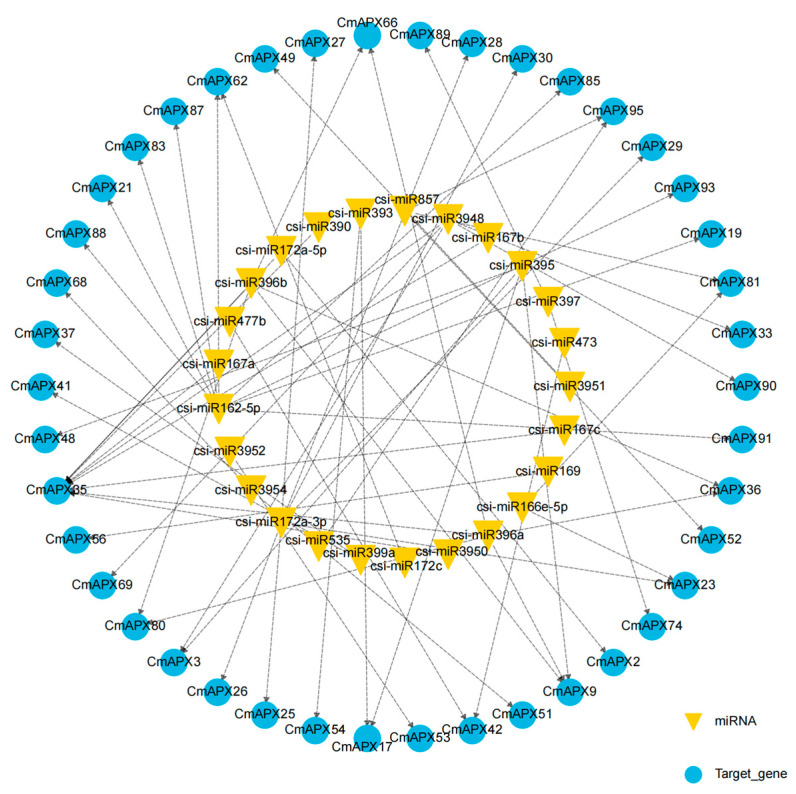
Network of miRNA–gene interaction of *APX* genes. Blue color represents the *APX* gene and yellow color represents the csi-miRNA associated with *APX*.

In order to investigate the potential regulatory effects of miRNAs on *APX* gene expression in pomelo, a total of 38 miRNAs were identified using the SapBase website and miRNA sequencing data for prediction, which may exert post-transcriptional regulatory effects on *APX* genes in pomelo [38]. The results indicate that the majority of miRNAs interact with multiple *CmAPX* genes. For instance, csi-miR162-5p and csi-miR172a-3p target the highest number of genes (7). Specifically, csi-miR162-5p simultaneously targets *CmAPX91*, *CmAPX93*, *CmAPX87*, *CmAPX85*, *CmAPX19*, *CmAPX21*, and *CmAPX83*, while csi-miR172a-3p targets *CmAPX23*, *CmAPX28*, *CmAPX29*, *CmAPX27*, *CmAPX26*, *CmAPX30*, and *CmAPX25*. Only three miRNAs, namely, csi-miR169, csi-miR3951, and csi-miR3952, target a single gene. Furthermore, *CmAPX35* can be regulated by multiple miRNAs (csi-miR167a, csi-miR167b, csi-miR167c, csi-miR172a-5p, csi-miR390, csi-miR3948, csi-miR3950, csi-miR396a, and csi-miR396b). Therefore, bioinformatics methods are crucial for predicting miRNA target genes in order to study miRNA functions and regulatory networks (Figure 7).

### 3.8. Expression Patterns of APX Gene during the Development of Pomelo Fruit Sacs

**Figure 8:**
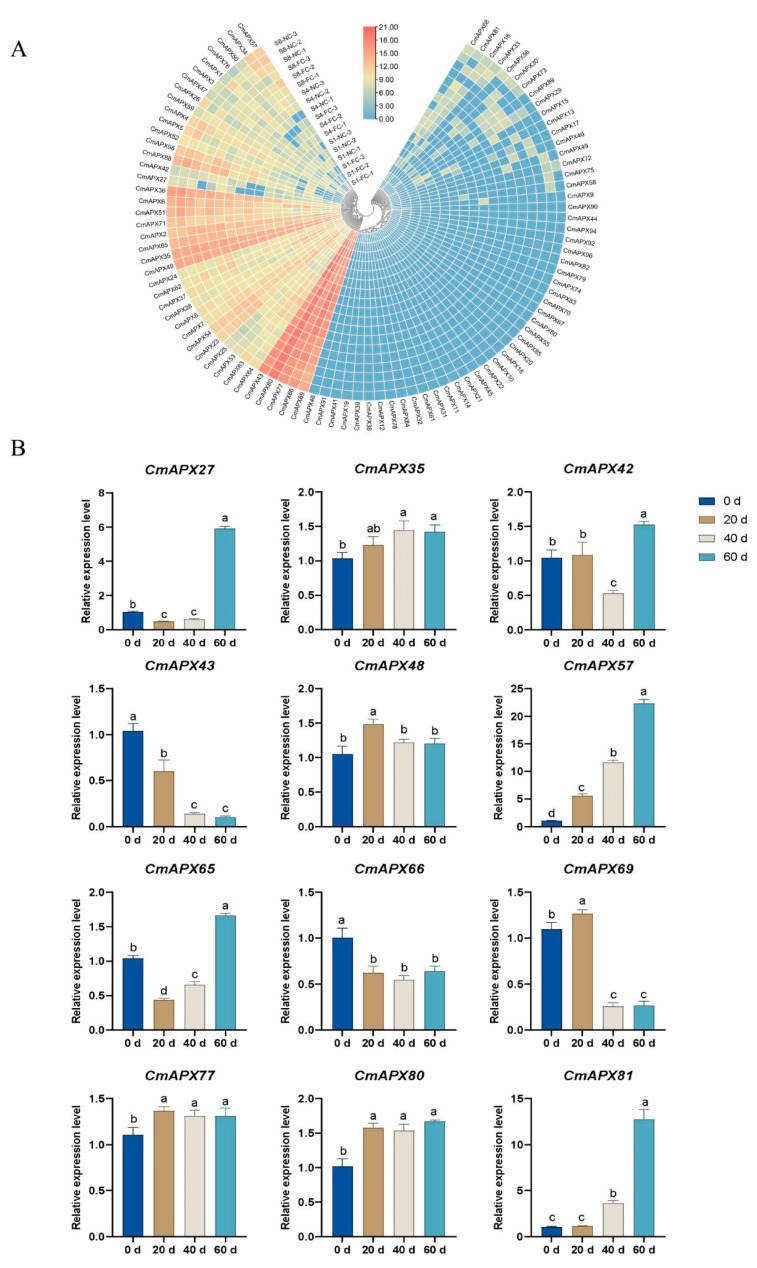
Expression profiling of *CmAPX* genes at different stages of development of pomelo fruit sacs (**A**) and under different storage times after harvest (**B**); one-way ANOVA was used to compare the expression level. Data represent mean values ± SD, n = 3. Different letters mean *p* < 0.05.

In order to analyze the expression pattern of *APXs* during the development of pomelo fruit sacs, FPKM values of pomelo fruit sacs near the core (NC) and far from the core (FC) were obtained from the transcriptome data (accession number PRJNA817805) and were selected for *APX* genes at 157, 180, and 212 days after flowering. The expression profiles of 93 *APX* genes were generated [36]. The results revealed differential expression levels of *APX* gene members across different growth stages of pomelo fruit. The 93 *CmAPX* genes were categorized into two groups: Group I comprised 41 *APX* members, including *CmAPX43*, *CmAPX80*, *CmAPX77*, *CmAPX66*, and *CmAPX69*, which exhibited high expression levels in both NC and FC stages across all three time points; other genes displayed varying patterns. Notably, the expression levels of *CmAPX57*, *CmAPX34*, *CmAPX50*, *CmAPX76*, *CmAPX1*, *CmAPX5*, and *CmAPX95* increased with bloom days in both NC and FC stages. Additionally, *CmAPX50*, *CmAPX76*, *CmAPX1*, *CmAPX42*, and *CmAPX27* showed significantly lower expression levels in the S4 stage compared to NC. Group II consisted of 52 genes, with 34 exhibiting low and unchanging expression levels in both NC and FC across all three stages. The expression levels of *CmAPX81* and *CmAPX16* are elevated during the S4-NC and S8-FC stages, while the expression levels of *CmAPX1*, *CmAPX42*, and *CmAPX27* are particularly heightened in the S8-FC stage (Figure 8A). These genes are potential candidate genes for future research on juice granulation during the postharvest storage of pomelo fruit.

### 3.9. Expression Analysis of APX in Pomelo Fruit under Different Storage Times after Harvest

Nie et al. (2020) report that pomelo fruit has a serious juice granulation problem during postharvest storage, which greatly reduces the attractiveness and commercial value of pomelo. The remission of juice granulation during the storage period of pomelo after harvest was attributed to the changes in the expression levels of various antioxidant enzymes at transcription and translation levels during AsA metabolism. In this study, qRT-PCR was utilized to analyze the differences in gene expression levels of *CmAPXs* at different storage periods (0 d, 20 d, 40 d, and 60 d) after fruit picking. As shown in Figure 8B, the expression levels of *CmAPX57* and *CmAPX81* increased significantly with the extension of storage time, which were about 12- and 6.5-fold of the expression levels of day 0, respectively. After 20, 40, and 60 days of storage, the expression levels of *CmAPX35*, *CmAPX48*, *CmAPX77*, and *CmAPX80* were slightly increased compared with day 0, and there was no significant difference between them. The expression levels of the *CmAPX43*, *CmAPX66*, and *CmAPX69* genes decreased significantly compared with day 0 and were about 5-, 2-, and 3.5-fold of the expression levels, respectively, which were significantly different from those before storage. In addition, *CmAPX27*, *CmAPX42*, and *CmAPX65* showed minor decreases at 20 or 40 days, followed by a significant decrease at 60 days.

As the structure of AsA is highly unstable, it is prone to degradation during respiration in fruits and vegetables, leading to easy loss during storage. The expression of *APX*, a key enzyme in the oxidative decomposition pathway of AsA, is closely associated with its content [39]. Therefore, the findings of this study indicate that the AsA content in pomelo fruit decreased with prolonged storage time, while there was a significant increase in the expressions of *CmAPX57* and *CmAPX81*, consistent with the pattern of the AsA oxidative decomposition pathway as revealed by transcriptome data.

## 4. Discussion

Pomelo is a highly storage-tolerant fruit. In this study, pomelos did not decay even after 60 days of postharvest storage, whereas tomatoes decay after 20 days [40]. The unique characteristics of pomelo ripening and aging may be due to the specificity of ROS metabolism, and APX plays a crucial role in ROS metabolism [41,42].

L-ascorbic acid (AsA), commonly known as vitamin C, is an essential compound for human health. AsA not only serves as a crucial antioxidant and scavenger of free radicals, particularly during plant photosynthesis and photoprotection, but also participates in cell growth and division and the biosynthesis of plant hormones [43]. Citrus fruits are widely acknowledged as one of the primary sources of vitamin C for human nutrition globally. The accumulation of AsA in citrus fruits is not only governed by intricate mechanisms but also influenced by changes in degradation and circulation rates. In particular, the variation in APX enzyme activity plays a crucial role in determining the AsA content in different fruits or within the same fruit during development or maturation [44,45].

The *APX* gene has been identified as a crucial gene in the degradation and regeneration pathways of AsA in various plant species. Research has identified 8, 8, 26, and 7 *APX* genes in *A. thaliana* [46], rice [47], cotton [48], and tomato [49], respectively, but they have rarely been reported in pomelo. In this study, a total of 96 *APX* gene family members were identified and designated as *CmAPX1*–*CmAPX96*. The abundance of these genes surpasses the number found in Arabidopsis, rice, tomato, and other species, possibly due to genome-wide replication events during the evolution of *APX* genes in pomelo. Discrepancies in the number of *APX* genes among different species may also stem from gene amplification or loss following whole-genome duplication events, as well as the intricate molecular mechanisms governing the ascorbic acid metabolism pathway and the presence of diverse regulatory mechanisms for *APX* genes [38].

The analysis of the physicochemical properties of *APX* genes in pomelo revealed that the complexity of the pomelo genome resulted in variations in the nucleotide sequence length of 96 *CmAPXs*. Additionally, differences in molecular weight and isoelectric points among members of the *CmAPX* family also indicated functional complexity. Furthermore, specific antioxidant enzyme genes are expressed in distinct plant organelles to efficiently eliminate H_2_O_2_ in different subcellular compartments. The surplus H_2_O_2_ in chloroplasts is primarily eliminated by *tAPX*, while mitochondrial *APX* actively participates in the detoxification of H_2_O_2_ generated by fatty acid β oxidation [50,51]. *OsAPX4* is localized in the peroxisome, and silencing *OsAPX4* can result in the premature aging of rice [52]. *CmAPX77*, closely related to *AtAPX3* and *OsAPX4*, specifically targets mitochondria in pomelo, facilitating its role in ROS clearance. Similarly, *AtAPX4* plays a significant role in H_2_O_2_ removal and enhancing plant salt tolerance. *CmAPX47*, homologous to *AtAPX4* and located in plastids, not only enhances eukaryotic photosynthesis but also serves as an important source for potential reactive oxygen species scavenging [53,54,55]. The photoprotective effect of *APX* is exclusive to eukaryotes following plastid acquisition, marking the evolutionary event linking ascorbic acid to antioxidant defense [56,57].

The amplification or loss of exons and introns can result in variations in gene architecture and functional differentiation [58,59]. The structural analysis of *CmAPX* genes revealed that genes within the same subpopulation or subbranch typically exhibit similar numbers of introns and conserved motif composition, indicating a high degree of conservation in intron position. This finding is consistent with the results reported by Pan et al. (2022) in cabbage, demonstrating that *BnaAPX* shares a similar gene structure to *AtAPX* across all subfamilies. Furthermore, the similarity in both the quantity and arrangement of exons (1–6) and introns (0–5) within the *CmAPX* gene suggests a non-random, highly conserved pattern of intron evolution in pomelo. Additionally, it is worth noting that *BnAPX* (Bra011683) consists of 10 exons, whereas *CmAPX87* and *CmAPX42* only have 2 exons, indicating variations in exon numbers among different plant species [60]. Strong collinearity was observed between pomelo and lemon, followed by citrus, and finally Arabidopsis and rice genomes. With the exception of three gene pairs with Ka/Ks values greater than 1, all other gene pairs had Ka/Ks values less than 1, indicating that the *CmAPX* gene may have undergone strong purifying selection pressure and segmental replication during the entire evolutionary process. These tandem replications and segmental duplications contributed to the expansion of new members and functions within the *APX* gene family during the evolution of the pomelo genome. This could enhance adaptability and evolution in pomelo organisms [61,62,63].

As a sessile organism, plants will activate their self-protection mechanism in response to various stresses, primarily through the upregulation of stress genes, and the expression of these genes is regulated by promoters and transcription factors. Therefore, the analysis of cis-acting elements within promoters plays a crucial role in studying the function of specific genes [64]. The analysis results of cis-acting elements revealed that the photoresponse elements were predominant in the *CmAPX* gene family, indicating a correlation between the functions of *CmAPX* gene family members and photoresponse, consistent with Fu et al. (2023). Ascorbate-related ROS-scavenging enzymes (SOD, APX), ascorbate regenerating enzyme (MDHAR, DHAR), and reduced glutathione regenerating enzyme (GR) exhibited significant responses to light intensity and light cycle during photodomestication, with APX activity playing a crucial role in maintaining ROS homeostasis in plants. The core promoter of a protein-coding gene typically contains a TATA-box, which serves as a cis-acting element and transcription binding site for specific protein regulation. It has been observed that all members of the *CmAPX* gene family contain the TATA-box [65]. In addition, members of the *CmAPX* gene family are also implicated in abiotic stress response, hormone regulation, and other functions. The irregular distribution of cis-acting elements in the promoter region facilitates the regulation of proteins under different environmental stimuli by being located in various positions.

Granulation is a physiological disorder of citrus fruits that occurs during maturation and postharvest storage, characterized by abnormal cell expansion, hardening, lignification of juice sacs, and loss of flavor. Typically, granulation first appears at the proximal end of the vesicle and progresses towards the core, with elongated juice sacs in the core being most severely affected [66]. During postharvest storage, affected juice sacs become dry and shriveled, often coexisting with preharvest granulation within the same fruit; in severe cases, the color of these cells turns earthy yellow. The spongy layer of the pericarp becomes loose and spongy, while there is no significant difference in appearance from normal fruit for the exocarp [67].

In order to comprehend the correlation between *APX*, active oxygen free radical scavenging, and cell granulation in pomelo juice sacs during development, we analyzed the transcriptome sequencing data of NC and FC juice sacs at different growth stages after flowering using the same method as reported by Li et al. (2022). The transcriptional levels of *CmAPX57*, *CmAPX34*, *CmAPX50*, *CmAPX4*, and *CmAPX5* exhibited a gradual increase towards the fruit core, while the expression level of *CmAPX81* was significantly elevated at the S4-NC stage, indicating an elevation in *APX* activity corresponding to an increased granulation degree. This may be attributed to the heightened intracellular reactive oxygen species resulting from cellular injury. Consequently, cells initiate an endogenous stress resistance system leading to enhanced *APX* enzyme activity, with the ultimate goal of combating damage caused by reactive oxygen species. Additionally, the expression levels of *CmAPX4* and *CmAPX5* were significantly lower in NC than FC at the S4 stage, whereas they exhibited an opposite trend at the S6 stage. This suggests that the expression of multiple *APX* isozyme genes in juice sacs varied significantly in response to oxidative stress during different developmental processes and granulation degrees. Furthermore, it indicates that fruit ripening involves growth and gradual aging, accompanied by an imbalanced accumulation of reactive oxygen species [13,68]. The expression of *CmAPX* serves to eliminate reactive oxygen species and plays a protective and coordinating role, the above results are consistent with the results of the GO enrichment analysis and qRT-PCR, and provide a scientific basis for reducing postharvest loss of pomelo fruit.

miRNAs, as a class of endogenous non-coding RNAs, play regulatory roles in eukaryotes. They achieve this by recognizing target miRNA through complementary base pairing and guiding the silencing complex to degrade the target miRNA or inhibit its translation. As a result, the spatio-temporal expression pattern of target genes is determined in coordination with transcriptional regulation guided by transcription factors [69]. In plants, post-transcriptional regulation mechanisms of miRNAs on target genes are widespread and play an important role in growth, development, and environmental response [70]. In this study, we discovered that a single miRNA has the ability to target multiple genes, while a gene can also be targeted by multiple miRNAs. Furthermore, the comprehensive mode of action of these miRNAs and *APX* in gene regulatory networks, as well as their interactions with key genes, remains poorly understood. Only 14 miRNAs targeting 33 *AhAPX* were identified in peanut, and 51 miRNAs targeting 29 *TaAPX* were identified in wheat. It was also observed that ath-miR447a-3p negatively regulates *APX3* in pepper and directly participates in the drought stress response of Zanthoxum [7,25,71]. These studies are highly significant for thoroughly analyzing the biological functions of miRNA in pomelo and further exploring miRNA gene resources in pomelo.

## 5. Conclusions

As one of the four types of citrus in the world, pomelo fruit is highly favored by consumers due to its large size, translucent flesh, abundant nutrients, distinctive flavor, and excellent storability and transportability. In this study, 96 *CmAPX* genes were identified from the pomelo genome, distributed randomly across nine chromosomes, exhibiting high conservation during evolution. The promoter sequences contained a significant number of hormone- and stress response-related cis-regulatory elements. The gene expression pattern analysis revealed that the relative expression levels of *CmAPX57* and *CmAPX81* increased with the degree of cell granulation intensification, indicating their potential role in reactive oxygen species scavenging and antioxidant activity, consistent with GO enrichment analysis results. Additionally, 38 miRNAs targeted 42 *CmAPX* genes. These findings lay a theoretical foundation for the further exploration of the functions of *CmAPX* genes in pomelo growth and development, abiotic stress responses, and hormone stresses.

## Data Availability

Data is contained within the article or Supplementary Materials.

## Supplementary Materials

The following supporting information can be downloaded at: https://www.mdpi.com/article/10.3390/genes15070911/s1, Table S1: The primers in this study; Table S2: Basic information of *CmAPX* gene; Table S3: The kaks analysis of *CmAPX* gene pairs.
